# An MRI-based radiomics nomogram for preoperative prediction of Ki-67 index in nasopharyngeal carcinoma: a two-center study

**DOI:** 10.3389/fonc.2024.1423304

**Published:** 2024-12-20

**Authors:** Yao Wang, Jing Zhang, Qiyuan Li, Li Sun, Yingmei Zheng, Chuanping Gao, Cheng Dong

**Affiliations:** ^1^ Department of Radiology, Affiliated Hospital of Qingdao University, Qingdao, China; ^2^ The Affiliated Hospital of Qingdao University, Qingdao, Shandong, China

**Keywords:** nasopharyngeal carcinoma, magnetic resonance imaging, Ki-67, radiomics, head and neck cancer

## Abstract

**Background:**

The expression level of Ki-67 in nasopharyngeal carcinoma (NPC) affects the prognosis and treatment options of patients. Our study developed and validated an MRI-based radiomics nomogram for preoperative evaluation of Ki-67 expression levels in nasopharyngeal carcinoma (NPC).

**Methods:**

In all, 133 patients with pathologically-confirmed (post-operatively) NPC who underwent MRI examination in one of two medical centers. Data from one medical center (n=105; Ki-67: ≥50% [n=57], <50% [n=48]) formed the training set, while data from another medical center (n=28; Ki-67: ≥50% [n=15], <50% [n=13]) formed the test set. Clinical data and routine MRI results were reviewed to determine significant predictive factors. The minimum absolute shrinkage and selection operator method was used to select key radiomics features to form a radiomics signatures from resonance imaging (MRI), and a radiomics score (Rad-score) was calculated. Subsequently, a radiomics nomogram was established using a logistic regression (LR) algorithm. The predictive performance of the nomogram was evaluated using operating characteristics curve (ROC), decision curve analysis (DCA), and the area under the curve (AUC).

**Results:**

Five radiomics features were selected to build the radiomics signature. The radiomics nomogram incorporating the clinical factors and radiomics signature showed favorable predictive value for expression level of Ki-67, with AUC 0.841 (95% confidence intervals: 0.654 –0.951) for the test set. Decision curve analysis showed that the nomogram outperformed a clinical model in terms of clinical usefulness.

**Conclusions:**

The radiomics nomogram based on MRI effectively predicted the pre-surgical expression level of Ki-67.

## Introduction

Nasopharyngeal carcinoma (NPC) is an epithelial carcinoma arising from the nasopharyngeal mucosal lining, and is the most common head and neck cancer. NPC is highly prevalent in southeast Asia and southern China (1–3). Of the 87 000 NPC cases newly diagnosed annually, over 70% are classified as advanced disease (4). 30%–40% of patients develop distant metastases within 4 years, and the response rate to radiotherapy for recurrent NPC is low, with 30%–50% of patients experiencing disease relapse after radical radio-chemotherapy (5, 6).

Ki-67 is a nuclear marker expressed in actively proliferating cells that is present in almost all phases of the cell cycle, except for the G0 phase, and accurately reflects the proliferative activity of cells. It is often used as a marker to assess the aggressiveness of tumors (7, 8). Previous studies reported that Ki-67 overexpression is an important marker of a poor prognosis in patients with NPC. Ki-67 expression levels can be used to plan radiotherapy and improve the patient prognosis with radiosensitizers (9, 10). Furthermore, previous research reports suggest that tumors with high levels of Ki-67 expression are more sensitive to radiation and exhibit a more pronounced response to radiation therapy (11). Comprehensive assessment of tumor aggressiveness facilitates individualized treatment and improves prognostic accuracy for patients with malignant tumors (12). Therefore, knowing the Ki-67 expression level is essential for planning radiotherapy and improving the patient prognosis. In nasopharyngeal carcinoma, Ki-67 expression can only be determined by immunohistochemistry through biopsy or surgical histopathology. However, this conventional test is invasive and requires the removal of tissue samples from the patient (11, 13), which involves a level of risk. Therefore, a non-invasive and accurate tool is urgently needed to more comprehensively and accurately predict Ki-67 expression in patients with NPC before they undergo surgery.

Radiomics is a new research methodology that allows the extraction of a large amount of imaging features in a high throughput manner (14). Radiomics can capture tissue and lesion characteristics, as well as heterogeneity across the entire tumor volume. Research has shown that radiomics features are closely related to the invasiveness of tumors and heterogeneity indices at the cellular level (15–17). Genomic analysis has shown that the degree of tumor heterogeneity is a prognostic factor for survival and a barrier to cancer control (18). Radiomics approaches have been successfully used to predict the Ki-67 index in various types of solid tumors, including breast cancer, non-small cell lung cancer, intrahepatic cholangiocarcinoma, glioma subtypes, and gastric cancer (19–23). However, to the best of our knowledge, radiomics analysis has not been used to predict the Ki-67 index in patients with NPC. The aims of this study were therefore to develop and validate an MRI-based radiomics nomogram that combines a radiomics signature with clinical factors for the preoperative prediction of Ki-67 index in patients with NPC.

## Methods

### Patients

This retrospective study included data from consecutive patients who received a histological diagnosis of NPC at one of two clinical centers between October 2015 and June 2023. This research only included patient image information. No other personal information about the patients was disclosed. Patients’ private information was adequately protected. The research was authorized by the ethical committees of the two medical centers and the requirement for informed consent was waived. The inclusion criteria for this study were as follows: (1) nasopharyngeal carcinoma confirmed by biopsy or surgery; (2) standard MRI scan within 14 days prior to treatment or biopsy; (3) detection of Ki-67 level by immunohistochemistry after surgery.

The exclusion criteria were as follows: (1) patients who underwent biopsy or surgery prior to MRI; (2) patients receiving preoperative chemoradiotherapeutic treatment; and (3) poor image quality.

**Figure 1:**
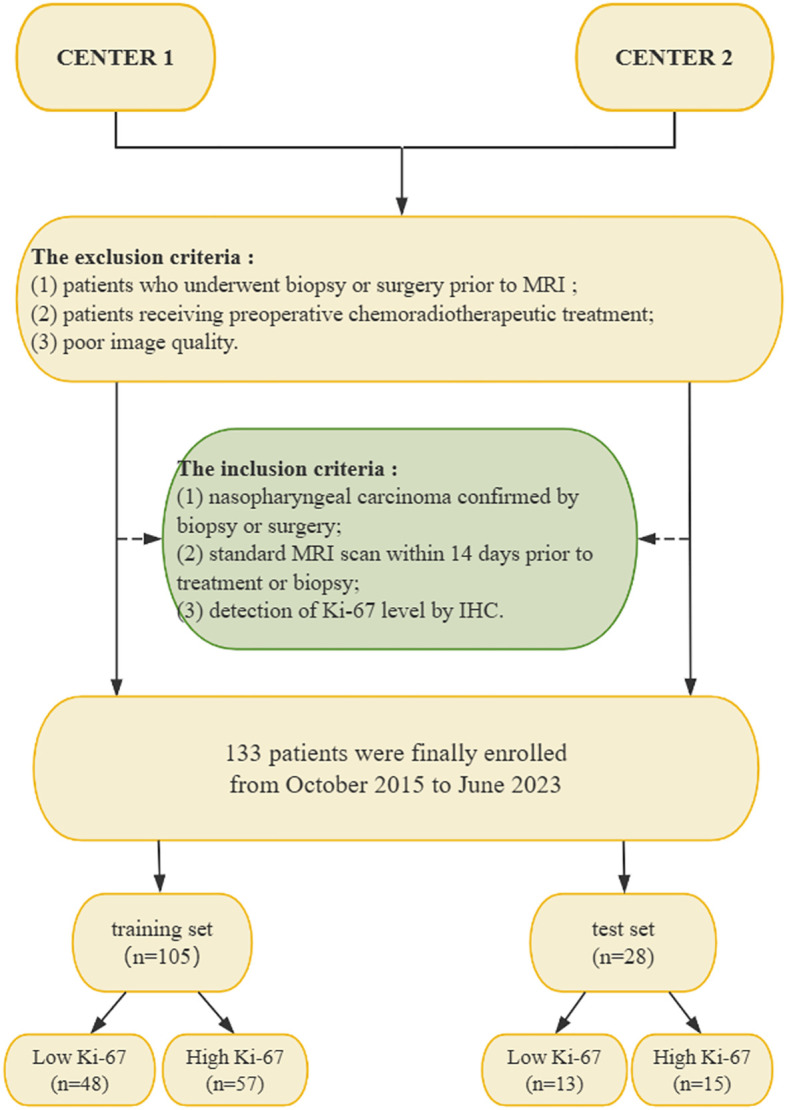
Flow chart of the patient recruitment pathway. NPC, nasopharyngeal carcinoma; IHC, immunohistochemistry; MRI, magnetic resonance imaging.

In total, 133 patients met the requirements of the study. 105 patients with NPC (74 men and 31 women; mean age, 51.29 ± 15.06 years; 57 high Ki-67 index and 48 low Ki-67 index) from one center were enrolled into a training set, and another cohort of 28 patients with NPC (18 men and 10 women; mean age, 49.54 ± 15.07 years; 15 high Ki-67 index, and 13 low Ki-67 index) from the second center were enrolled into an external test set. **Figure 1** illustrates the patient recruitment pathway.

### Ki-67 index measurement

In this study, Ki-67 indices were determined for all 217 patients through immunohistochemistry (IHC) performed on surgical histopathology samples. The IHC staining was carried out using a Ki-67 protein antibody (Santa Cruz Biotechnology). Cells exhibiting brown-stained nuclei were considered positive for Ki-67 expression. The Ki-67 index was calculated as the percentage of Ki-67-positive cells among 1,000 randomly selected cells, which were visualized at 200× magnification. According to previous studies, if the Ki-67 index was ≥ 50%, we considered the Ki-67 level to be high expression, whereas if the index was < 50%, we considered the Ki-67 level to be low expression (11, 23). Indeed, the median Ki-67 index for our training set was found to be 50%. Two pathologists (with 4 and 8 years of experience) interpreted the Ki-67 indices in consensus.

### MR image acquisition

For all patients, the preoperative MRI included axial T1-weighted imaging (T1WI) and fat-suppressed T2-weighted imaging (FS-T2WI) acquired using a General electric Signa HDX 3.0-T MRI scanner. The repetition time (TR) and echo time (TE) for the 3.0-T MRI scans were 600/15 ms, respectively, for T1WI, and 300085/100 ms for FS-T2WI. For both sequences, the slice thickness was 4 mm, the interlayer spacing was 1.0 mm, the matrix was 256 × 512, and the field of view was 200 × 200 mm.

### Analysis of conventional MRI findings

Axial T1WI and axial FS-T2WI extracted from an image archiving and communication system were used for feature extraction. Retrospective independent assessment was performed by two radiologists (radiologist A with 8 years of experience in diagnosing NPC and radiologist B with 16 years of experience) who were unaware of the pathological diagnosis. The evaluated MRI features included maximum tumor length (the maximum diameter of the largest cross-section of the tumor), tumor necrosis (a significant hyperintense area on FS-T2WI), surrounding tissue spread (involvement of tissues other than nasopharynx), lymphatic spread (short axial diameter cervical lymph nodes of > 10 mm), and lymphatic necrosis (a significant hyperintense area on FS-T2WI).

### Construction of the clinical model

Differences in clinical factors (including clinical data and conventional MRI findings) between high and low Ki-67 expression groups in the training set were compared using chi-square and independent samples t-test analyses. Specific clinical factors of concern include gender, age, maximum tumor length (the maximum diameter of the largest cross-section of the tumor), tumor necrosis (a significant hyperintense area on FS-T2WI), surrounding tissue spread (involvement of tissues other than nasopharynx), lymphatic spread (short axial diameter cervical lymph nodes of > 10 mm), and lymphatic necrosis (a significant hyperintense area on FS-T2WI), as shown in **Table 1**. Multiple logistic regression analysis was used to build a clinical model based on significant variables identified in the univariate analysis.

**Table 1:** Clinical factors of the training and test sets.

Clinical factors	Training set (n=105)			Test set (n=28)		
	Low Ki-67 index (Ki-67 < 50%, n=48)	High Ki-67 index (Ki-67 ≥ 50%, n=57)	*P1*	Low Ki-67 index (Ki-67 < 50%,n=13)	High Ki-67 index (Ki-67 ≥ 50%,n=15)	*P2*
Gender (M/F)	32/16	42/15	0.128	8/5	10/5	0.787
Age, year	53.02 ± 14.24	49.82 ± 15.68	0.133	51.46 ± 13.99	47.87 ± 16.24	0.539
Maximum tumor length(mm)	31.29 ± 12.83	31.04 ± 11.98	0.595	32.00 ± 11.61	31.67 ± 11.81	0.941
Tumor Necrosis (Absent/Present)	22/26	19/38	0.191	7/6	13/2	0.096*
Surrounding tissue spread (Absent/Present)	24/24	36/21	0.175	8/5	11/4	0.689*
Lymphatic spread (Absent/Present)	25/23	11/46	<0.001	7/6	4/11	0.142
Lymphatic Necrosis (Absent/Present)	33/15	20/37	0.001	8/5	11/4	0.689*

Numerical data are presented as mean± standard deviation, categorical data as numbers (n).

F: female; M: male.

*P1*: the *P* value of comparison between low Ki-67 index group and high Ki-67 index group in training set; *P2*: the *P* value of comparison between low Ki-67 index group and high Ki-67 index group in test set.

*: Fisher’s exact test.

### Image segmentation and radiomics feature extraction

After obtaining the MRI data, the two radiologists used 3D Slicer software (version 4.10.2, https://www.slicer.org/) to perform 3D segmentation of the lesion region of interest (ROI) for extraction of radiomics features. The radiologists manually traced the ROI within the tumor boundary on each tumor plane of the axial T1WI, using the axial fat-suppressed T2WI as an aid. The two radiologists were blinded to the patients’ clinical results while performing this segmentation procedure. Adjacent normal tissue and vessels were not included in the ROI. Considering the different protocol parameters of the different MRI scanners that would be used in clinical practice, a series of preprocessing procedures were applied. The “μ ± 3σ” method was used to correct for the influences of MRI scanners and acquisition protocols, and the image intensity was normalized (24). At the same time, N4ITK bias correction was used to correct for intensity non-uniformity caused by non-uniformity of the scanner magnetic field during image acquisition (25).

**Figure 2:**
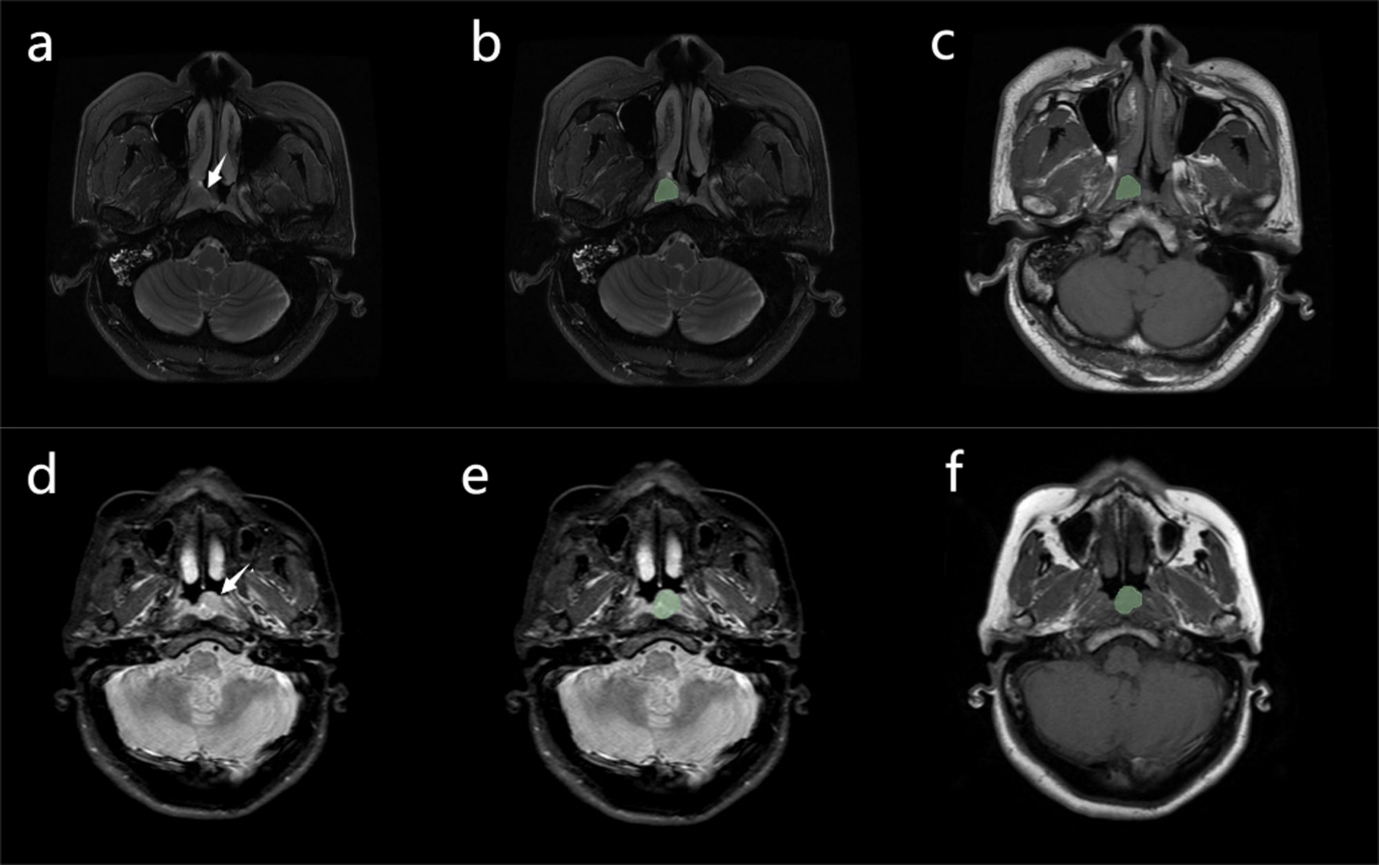
**(A)** Case 1: nasopharyngeal carcinoma in a 52-year-old man, with Ki-67 expression measured at 30% by immunohistochemistry. A mass can be seen in the nasopharynx (arrow). **(B, C)** Manual segmentation of the mass. Conventional magnetic resonance imaging of the patient showed no signs of cervical lymphatic metastases and lymphatic necrosis. The values of radiomics features (A to E, presented in **Table 2**) were 0.092, 1.099, 0.384, -0.026, and-0.244, respectively. The Rad-score was -1.413, the Nomo-score was -2.431 **(D)** Case 2: nasopharyngeal carcinoma in a 49-year-old man, with Ki-67 expression measured at 65% by immunohistochemistry. A mass can be seen in the nasopharynx (arrow). **(E, F)** Manual segmentation of the mass. The patient’s conventional MRI showed cervical lymphatic metastases with no evidence of lymphatic necrosis. The values of radiomics features (A to E, presented in **Table 2**) were 1.665, 0.861, 1.345, -0.015, and 0.790, respectively. The Rad-score was 1.796, the Nomo-score was 1.772.

To guarantee the repeatability of the results, resampling and z-score normalization were performed as preprocessing steps for images and data, respectively. In the first step, the MR images were resampled to a voxel size of 1 × 1 × 1 mm to ensure scale conservation when deriving 3D features. Feature extraction was performed using the Slicerradiomics model in the 3D Slicer radiomics Extension Pack (v.4.10.2 https://www.slicer.org/). The radiomics features were then extracted using the lesion ROI delineated on the original MR images (**Figure 2**). The radiomic features extracted from T1WI and FS-T2WI respectively include the following: shape (n=14), gray level dependence matrix (GLDM, n=14), gray level co-occurrence matrix (GLCM, n=24), first-order statistics (n=18), gray level run length matrix (GLRLM, n=16), gray level size zone matrix (GLSZM, n=16), neighboring gray tone difference matrix (NGTDM, n=5), and wavelet (n=744) features. In total, 1702 radiomics features were extracted from each patient’s tumor (**Supplementary Material**).

### Intraobserver and interobserver reliability of the radiomics features

23 cases in the training set were randomly selected to calculate the interclass and intraclass correlation coefficients (ICC) for the radiomics features. ROI segmentation was conducted independently by Radiologists A and B during the same period to allow assessment of inter-observer agreement in the extracted radiomics features. Radiologist A again segmented these cases at 7-day intervals to assess intraobserver reliability. Features with good agreement were defined as those with an ICC > 0.75, and were forwarded to a further feature selection process. Radiologist A then performed the radiomics extraction of these features on the remaining samples.

### Feature selection and development of the radiomics signature

A two-step method was used to perform the further feature selection on the training set data. First, features with interobserver and intraobserver ICCs >0.75 were tested by one-way analysis of variance (ANOVA) to identify those showing significant differences between the high and low Ki-67 index. The selected features that showed significant differences were then entered into a least absolute shrinkage and selection operator (LASSO) regression model to obtain the features (non-zero coefficients) that were most valuable for predicting the Ki-67 index in the training set. The radiomics features selected in this procedure were those with non-zero coefficients in a 10-fold cross-validation where the regularization parameter (λ) was adjusted to control the regularization strength through the minimum criterion for a simple model. A radiomics score (Rad-score) was then calculated for each patient (in both the training and test sets) via the logistic regression product of the features weighted by their respective coefficients.

### Development of the radiomics nomogram and assessment of the performance of the different models

The independent clinical factors were combined with the rad-score using multiple logistic regression to construct a radiomics nomogram. In this manner, a radiomics nomogram score (Nomo-score) was calculated for each patient in the training and test sets.

Calibration curves were used to show the performance of the nomogram graphically (evaluating the agreement between the predicted and actual Ki-67 probabilities), and the data from the test set were used to verify the nomogram’s validity.

The area under the curve (AUC) was used to evaluate the performance of the three models (clinical model, radiomics signature, and radiomics nomogram) on the training and test sets, and the DeLong test was used to compare prediction performance between them. For both training and test sets, accuracy, sensitivity, specificity, and their 95% confidence intervals (CI) were calculated for each model. Decision curve analysis (DCA) was used to assess the usability and efficiency of the three models.

### Statistical analysis

SPSS software (version 25.0), MedCalc software (version 11.4.2.) and R software (version 3.3.3) were used for statistical analysis. Continuous variables are expressed as mean ± standard deviation and were compared between patient groups using independent samples *t*-tests. P < 0.05 was regarded as statistically significant. Qualitative data were compared using the chi-square test or Fisher’s exact test. One-way ANOVA was used to compare the values of different radiomics characteristics between the high and low Ki-67 groups. DCA quantifies the “net benefit” of the three models when applied to the test set at different threshold probabilities, with this analysis including the ability to graphically display the net benefit of the radiomics models. ROC analysis was performed using Medcalc. Nomogram, calibration curve, decision curve, and LASSO logistic regression analyses were performed using the R “rmc”, “rmda” and “glmnet” software packages.

## Results

### Clinical model construction

**Table 1** provides details of the clinical data and MRI features in the training and test sets. Lymphatic necrosis and lymphatic spread showed significant differences between the high and low Ki-67 index groups in the training set. The other clinical factors (including clinical data and conventional MRI findings) did not show statistically significant differences between the high and low Ki-67 index groups (P > 0.05). After multivariate logistic regression analysis, lymphatic necrosis (p = 0.035, OR = 2.860, CI, 1.075–7.613) and lymphatic spread (p = 0.048, OR = 2.549, CI, 1.009–6.440) were identified as independent predictors of Ki-67 and were used to construct the clinical model. A high Ki-67 index was more common in patients with NPC with lymphatic necrosis or lymphatic spread.

### Feature selection and development of the radiomics signature

**Figure 3:**
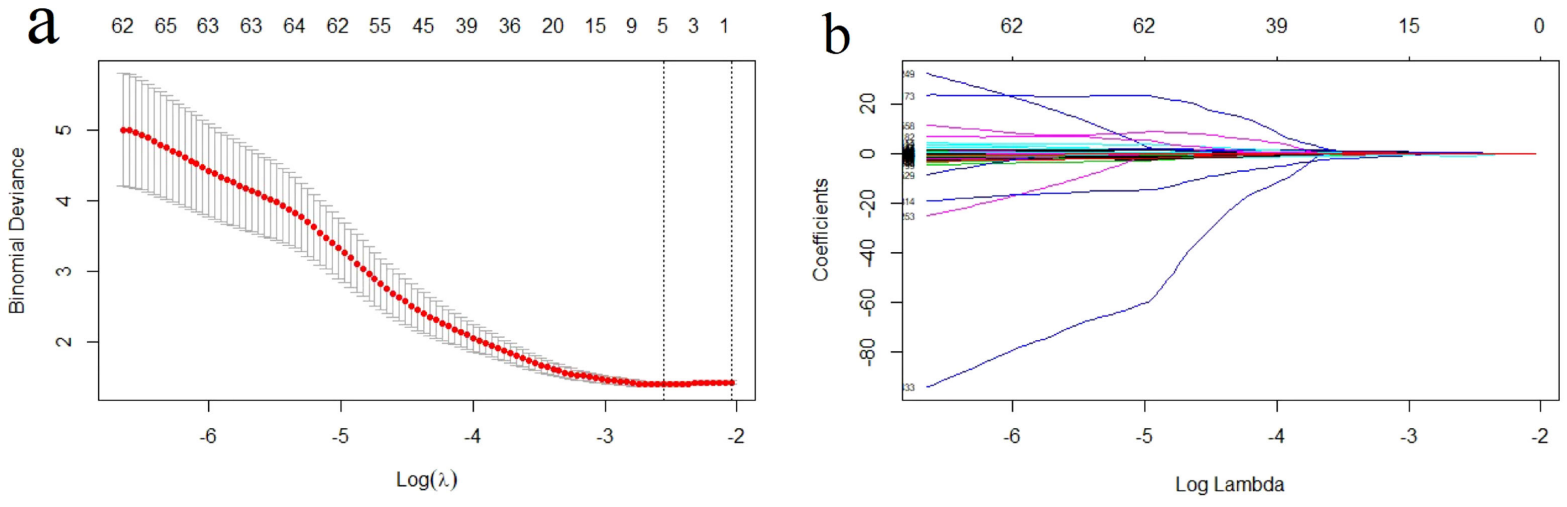
Radiomics feature selection using the least absolute shrinkage and selection operator (LASSO) regression model. **(A)** Tuning parameter (λ) selection in LASSO model used a 10-fold cross-validation via minimum criterion. The optimal values of the LASSO tuning parameter (λ) are indicated by the dotted vertical lines, and a value λ of 0.078 was chosen. **(B)** LASSO coefficient profiles of the 572 radiomics features. A coefficient profile plot was generated versus the selected log λ value using 10-fold cross-validation. Five radiomics features with non-zero coefficients were selected.

A total of 1526 radiological features with an ICC > 0.75 were considered in the one-way ANOVA for further selection. In this one-way ANOVA, 572 features showed statistically significant differences between the high and low Ki-67 index groups in the training set. Finally, the LASSO regression with λ of 0.078 identified five features with non-zero coefficients that were used to develop the Rad-score (**Figure 3**).

Using these features, the Rad-score was calculated as follows: Rad-score = -4.388+(1.202 × A) + (2.451 × B) + (1.202 × C) + (4.884× D) +(0.67 × E). The variables A to E represent the selected radiomics features, as shown in **Table 2**. An independent samples *t*-test showed a significant difference in Rad-score between the high Ki-67 index group and the low Ki-67 index group in the training set (**Table 3**).

**Table 2:** Radiomics feature selection results.

Variables	Radiomics feature name	Sequence
A	wavelet-LHL. glcm. Correlation	FS-T2WI
B	wavelet-LHH. glcm. InverseVariance	FS-T2WI
C	wavelet-HLH. gldm. LowGrayLevelEmphasis	FS-T2WI
D	wavelet-HHH.firstorder. Median	FS-T2WI
E	wavelet-LLL. firstorder. Skewness	FS-T2WI
Rad-score = -4.388+(1.202 × A) + (2.451 × B) + (1.202 × C) + (4.884× D) +(0.67 × E)		

GLCM, gray level co-occurrence matrix; GLDM, gray level dependence matrix.

Rad-score: radiomics score.

**Table 3:** The results of Rad-score and Nomo-score in the training and test sets.

	Training set (n = 105)			Test set (n =28)		
	Low Ki-67 index (Ki-67 < 50%, n=48)	High Ki-67 index (Ki-67 ≥ 50%, n=57)	*P1*	Low Ki-67 index (Ki-67 < 50%,n=13)	High Ki-67 index (Ki-67 ≥ 50%,n=15)	*P2*
Rad-score	-1.142 ± 3.701	1.552 ± 5.221	0.003	-0.586 ± 0.921	0.689 ± 0.819	0.001
Nomo-score	-1.364 ± 2.842	1.887 ± 5.392	< 0.001	-0.790 ± 0.993	0.598 ± 1.312	0.004

Numerical data are presented as mean ± standard deviation, categorical data as numbers (n).

Rad-score, radiomics score; Nomo-score, nomogram score.

P1: the p value of comparison between high Ki-67 index group and low Ki-67 index group in training set; P2: the p value of comparison between high Ki-67 index group and low Ki-67 index group in test set.

### Development of the radiomics nomogram

**Figure 4:**
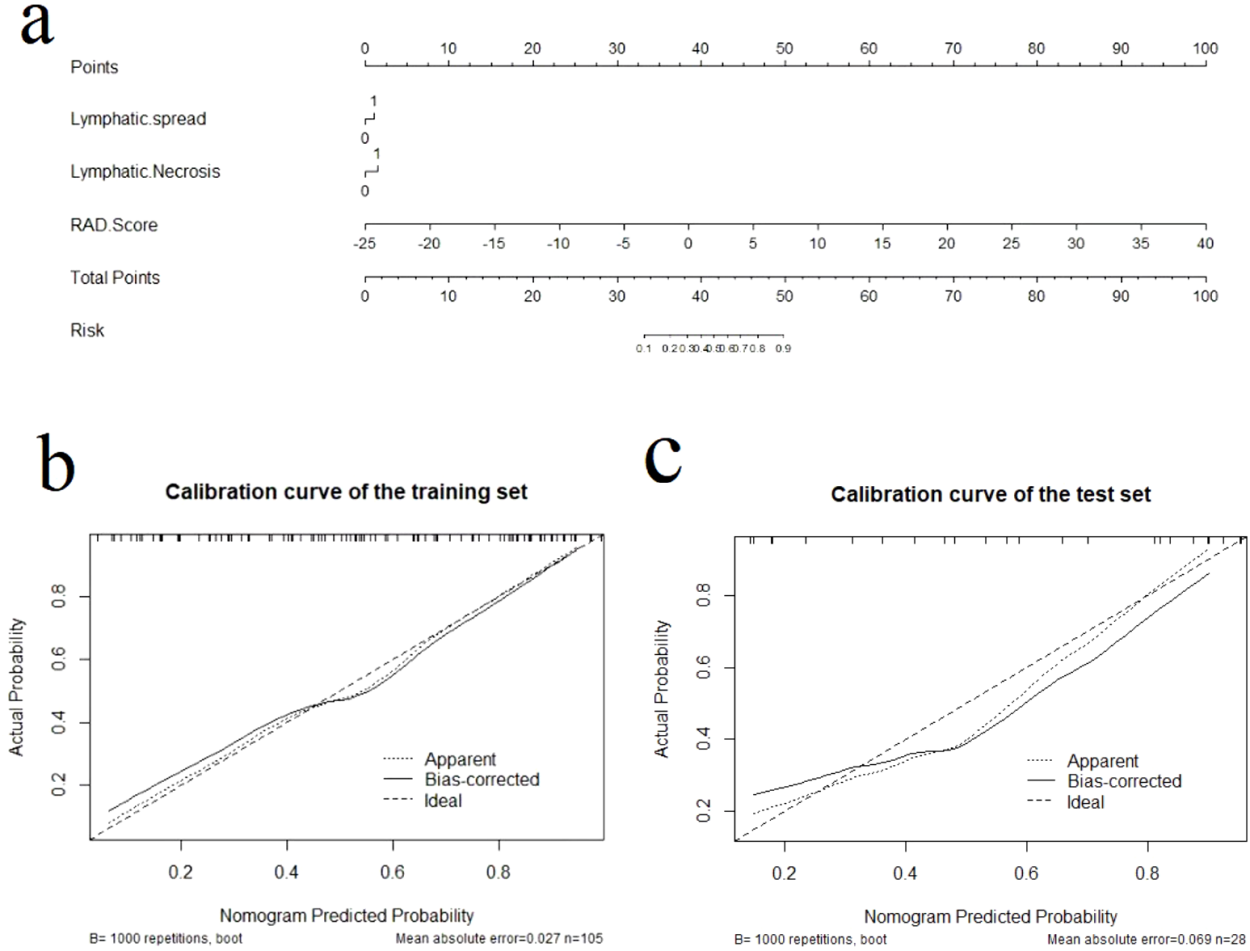
The radiomics nomogram and calibration curves for the radiomics nomogram. **(A)** The radiomics nomogram developed using the training set, combining lymphatic necrosis, lymphatic spread, and radiomics score. Calibration curves for the radiomics nomogram in the training **(B)** and test **(C)** sets. Calibration curves indicate the goodness-of-fit of the nomogram. The 45° straight line represents the perfect match between the actual (Y-axis) and nomogram-predicted (X-axis) probabilities. A closer distance between two curves indicates higher accuracy.

We used multifactorial logistic regression to construct a radiomics nomogram combining rad-score, lymphatic necrosis, and lymphatic metastasis (**Figure 4A**). The calibration curves for this are shown in **Figures 4B, C**. The calibration curve showed a good calibration effect in the training set. The Nomo-score for this nomogram was then calculated using the formula: Nomo-score = −0.922 + (1.067 × lymphatic necrosis) + (0.776 × lymphatic spread) + (1.068 × Rad-score). In the training set, the Nomo-score showed a statistically significant difference between the high and low Ki-67 index groups (**Table 3**).

### Assessment of the performance of the different models

**Figure 5:**
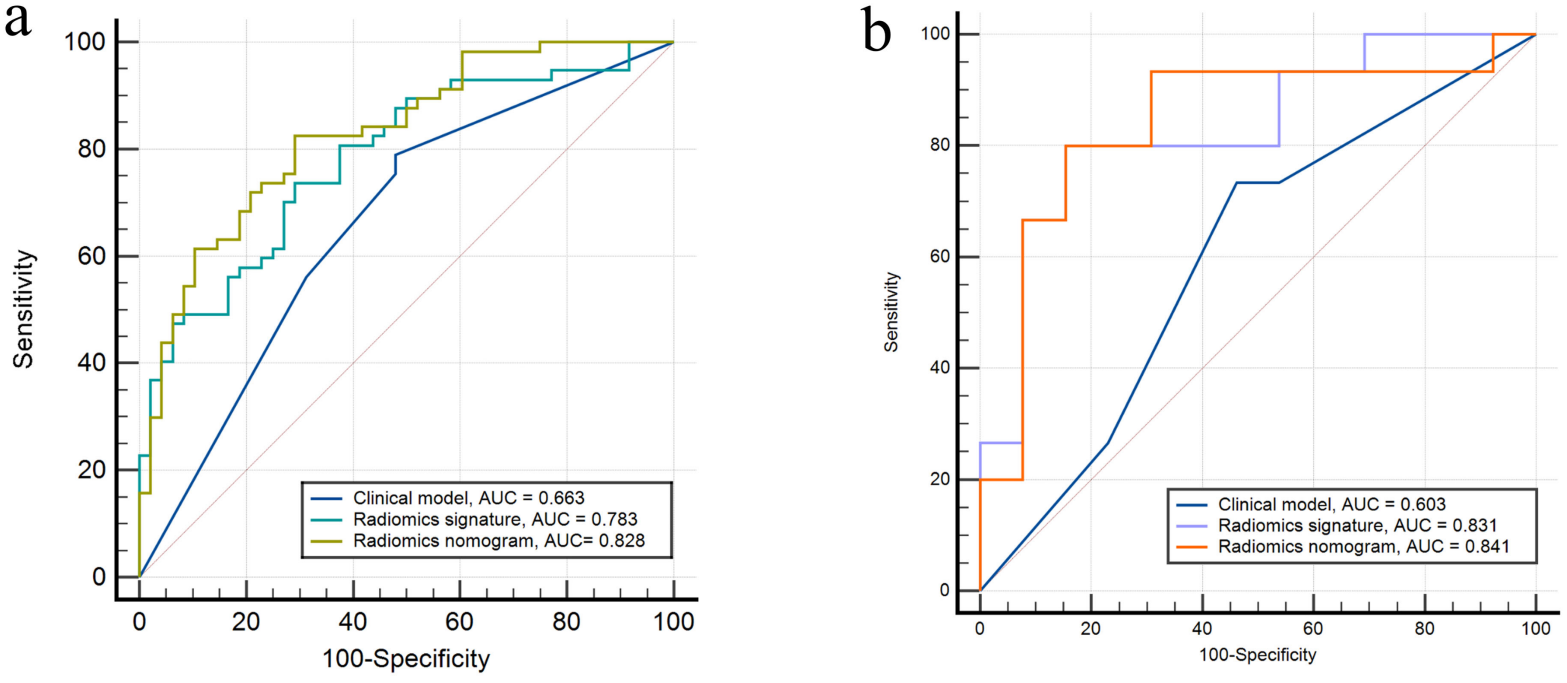
Receiver operating characteristics curves of the three models in the **(A)** training and **(B)** test sets, respectively.

**Figure 6:**
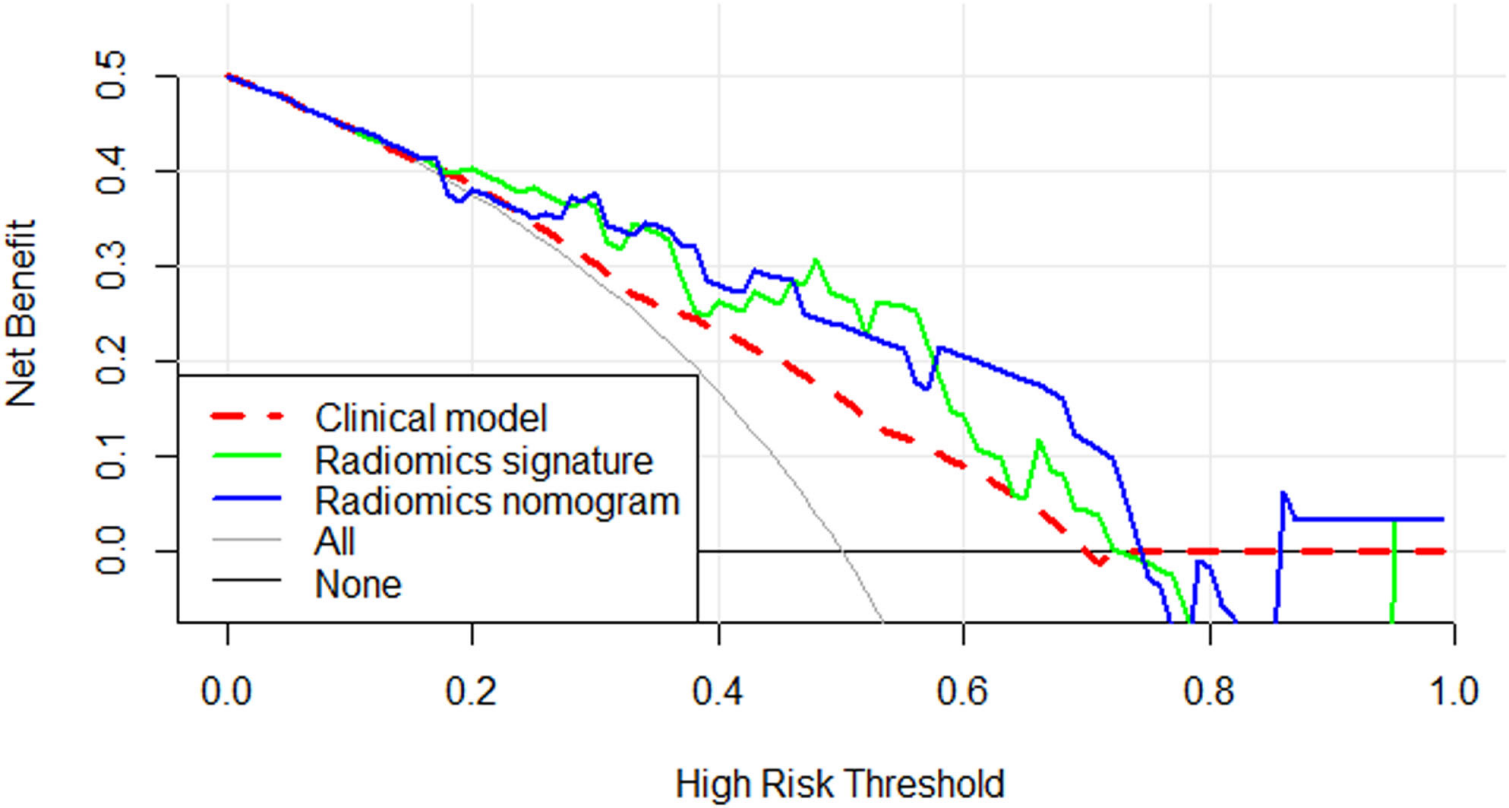
Decision curve analysis for the three models. The y-axis indicates the net benefit and the x-axis indicates threshold probability. Both the radiomics signature and radiomics nomogram had a higher overall net benefit for differentiating high Ki-67 index from low Ki-67 index than the clinical factor model and simple diagnoses such as all high Ki-67 patients (gray line) or all low Ki-67 patients (black line). This was the case across the full range of threshold probabilities at which a patient with NPC would have a high Ki-67 index.

Calibration curves for the different models are shown in **Figures 4B, C**. **Table 4** presents the AUC, sensitivity, specificity, and accuracy of each model. **Figure 5** shows the ROC curves of each model for the training and test sets. The radiomics nomogram achieved AUC values of 0.828 (0.742–0.895) and 0.841 (0.654 –0.951) in the training and test sets, respectively. The Delong test indicated that the radiomics nomogram predicted Ki-67 expression better than the clinical factors in the training set (AUC: 0.828 vs 0.663, p = 0.0002) and the test set (AUC: 0.841 vs 0.603, p = 0.0257). According to the DCA results, within a reasonable threshold probability range, the radiomics nomogram for predicting Ki-67 expression in patients with NPC had higher net benefits than the clinical factor model (**Figure 6**).

**Table 4:** Diagnostic performance of the clinical factor model, the radiomics signature, and the radiomics nomogram.

Model	AUC (95%CI)	Sensitivity %(95%CI)	Specificity %(95%CI)	Accuracy %(95%CI)
Training set (n=105)				
Clinical model	0.663 (0.565 to 0.753)	78.95 (66.1 to 88.6, 45/57)	52.08 (37.2 to 66.7, 25/48)	66.67 (57.6 to75.7, 70/105)
Radiomics signature	0.783 (0.692 to 0.857)	73.68 (60.3 to 84.5, 42/57)	70.83 (55.9 to 83.0, 34/48)	72.38 (63.8 to 80.9, 76/105)
Radiomics nomogram	0.828 (0.742 to 0.895)	82.46 (70.1 to 91.3, 47/57)	70.83 (55.9 to 83.0, 34/48)	77.14 (69.1 to 85.2, 81/105)
Test set (n=28)				
Clinical model	0.603 (0.401 to 0.781)	73.33 (44.9 to 92.2, 11/15)	53.85 (25.1 to 80.8, 7/13)	64.29 (46.5 to 82.0, 18/28)
Radiomics signature	0.831 (0.642 to 0.945)	80.00 (51.9 - 95.7, 12/15)	84.62 (54.6 - 98.1, 11/13)	82.14 (68.0 to 96.3, 23/28)
Radiomics nomogram	0.841 (0.654 to 0.951)	80.00 (51.9 - 95.7, 12/15)	84.62 (54.6 - 98.1, 11/13)	82.14 (68.0 to 96.3, 23/28)

CI, Confidence interval. Data in the parentheses are raw data.

## Discussion

In this study, we developed and validated a non-invasive MRI radiomics nomogram composed of radiomics features combined with clinical parameters to predict the Ki-67 index in individual patients with NPC. Our radiomics nomogram performed well in the prediction of Ki-67 index (AUC: test set, 0.841). Calibration curves and decision curve analysis (DCA) showed good fitness and high clinical utility. At the time of the patient’s visit, an MRI scan was performed, lymphatic necrosis and lymphatic spread were recorded. The selected radiomic features (as shown in **Table 2**) were extracted from the lesion ROI delineated on the original MRI images to calculate the Rad-score. The Nomo-score was then derived using the following formula: Nomo-score = -0.922 + (1.067 × lymphatic necrosis) + (0.776 × lymphatic spread) + (1.068 × Rad score), which predicts Ki-67 expression levels in NPC patients. Therefore, subject to further validation, our model could be used as a non-invasive, preoperative, and effective diagnostic method for treatment planning and prognosis assessment in patients with NPC. Our research is important for finding new methods to treat NPC.

For this study, we selected axial T1WI and axial FS-T2WI as MRI sequences. MRI provides better soft tissue resolution than CT, allowing for improved differentiation between lymph nodes and adjacent primary tumors. It is common for NPC to invade the skull base during diagnosis, and the apexes of the slope, wing, sphenoid body, and petrous temporal bone are often affected. In this respect, axial T1WI allows effective assessment of the degree of skull base invasion (26). FS-T2WI is a valuable tool for evaluating tumors, it helps to assess their location, size, shape, growth pattern, and extent, and is particularly effective for detecting necrotic cystic changes within tumors (27).

NPC has various biomarkers, among which the expression of Ki-67 is associated with the aggressiveness and prognosis of NPC. Zhang et al. (28) reported that Ki-67 expression level was significantly correlated with progression-free survival, and Chang et al. (29) found that Ki-67 expression was related to the survival rate of patients with NPC. Furthermore, it is reported that Ki-67 overexpression was associated with poor overall survival in Asian NPC patients, with cutoff values ≥50% (30). Notably, there are currently no standardized criteria for classifying Ki-67 expression levels in NPC patients. Previous studies have used the median as a cutoff point (9, 11, 31). In this study, since the median Ki-67 index in the training set is 50%, we defined a high Ki-67 index as that of patients with a Ki-67 index of 50% or higher. Our research results indicate that the radiomics nomogram can effectively predict the expression of Ki-67. Moreover, the radiomics nomogram could potentially be a valuable tool for evaluating the patient prognosis and formulating personalized treatment plans.

In this study, we assessed the value of clinical data (age and sex) and imaging data (maximum tumor length, tumor necrosis, surrounding tissue spread, lymphatic metastasis and lymphatic necrosis) for predicting Ki-67 index. Our study of NPC revealed a strong association between the expression of Ki-67 and cervical lymphatic metastasis, and also between Ki-67 and lymphatic necrosis (p < 0.05). Lymphatic necrosis is a significant imaging feature of malignant lymph nodes; because of the rich lymphatic network in the nasopharynx, NPC often metastasizes to the lymph nodes. Tang et al (32) reported that retropharyngeal lymphatic metastasis affects the survival of NPC patients. Huang et al (33) reported that the maximum axial diameter of metastatic lymph nodes (4 cm) is a significant adverse prognostic factor for overall survival. According to Ying et al., patients with larger areas of lymph node necrosis have a higher risk of death, local recurrence, and local and distant metastasis after radiotherapy than those without lymph node necrosis (34). The Ki-67 index reflects the heterogeneity and invasiveness of the tumor (12), and as noted previously, those patients with NPC who have a high Ki-67 index have a worse prognosis. Our research results also indicate that patients with NPC with lymphatic necrosis and lymphatic metastasis are more likely to exhibit a high Ki-67 index.

Radiomics is a recent imaging method that involves extracting detailed radiomic features from medical images (35). Radiomics can be used to capture the characteristics of tissues and lesions, characteristics that may be associated with the invasiveness of tumors. Radiomics approaches have also been used to predict clinical endpoints such as survival and treatment response and were shown to be directly related to proteomic features (15, 18). Radiomics analysis has shown good diagnostic performance when used to predict the Ki-67 expression level in various tumors. Qian et al (21) developed a radiomics model with preoperative enhanced MR images to predict the expression status of Ki-67 in intrahepatic cholangiocarcinoma (AUC = 0.815). Liang et al (35) developed a radiomics classifier based on T2WI (AUC = 0.740) to predict high Ki-67 status in breast cancer patients. Another study successfully developed a radiomics signature for predicting the Ki-67 index of meningiomas (AUC = 0.819) (36). The study of Fan et al. demonstrated that an MRI-based radiomics model was useful for predicting Ki-67 expression level in hepatocellular carcinoma (AUC = 0.863) (37). These previous studies suggest that MRI-based radiomics features have the potential to predict Ki-67 expression levels in NPC, and our results indicate that the MRI-based radiomics nomogram developed in this study showed good performance in predicting the Ki-67 index in patients with NPC, with an AUC of 0.841 in the test set. In our study, we used LASSO logistic regression to identify five radiomics features that we then used to develop a radiomics signature model. These features were wavelet-filtered features from FS-T2WI images. MRI FS-T2WI sequences not only clearly show the morphology of the lesions, but also reflect the pathological features of the lesion tissue (38). The addition of inter-tissue contrast may allows the images to contain radiomics features that are more reflective of tissue heterogeneity. The wavelet transformed features are obtained through wavelet decomposition of the first-order and texture features, and can extract intratumor heterogeneity information from the original images. The wavelet features selected by our radiomics model were GLCM, GLDM, and first-order statistical features, which can reflect texture heterogeneity and tend to be correlated with tumor heterogeneity (39). Research demonstrated that radiomic features are significantly associated with heterogeneity indices at the cellular level, and the extent of tumor heterogeneity is a key factor in determining prognosis (18). The Ki-67 index is linked to the prognosis of patients with NPC, which may explain why radiomic features reflecting tumor heterogeneity can predict the Ki-67 expression level in patients with NPC.

This study had certain limitations. First, selection bias in retrospective studies, even with strict inclusion and exclusion criteria, was impossible to completely eliminate. Second, manual segmentation was used to determine the ROI of all tumors, which may introduce bias. To more accurately represent the ROI, a time-consuming but reliable automatic segmentation method should be developed in future studies. In addition, further prospective studies are needed to more accurately predict the prognosis of nasopharyngeal carcinoma patients. Finally, only 133 patients were included in this study, which constitutes a relatively small study population.

## Conclusions

Our MRI-based radiomics nomogram performed well in the prediction of Ki-67 index (≥ 50% vs < 50%) in patients with NPC. It may be useful for prognostication and clinical decision-making in patients with NPC.

## Data Availability

The original contributions presented in the study are included in the article/**Supplementary Material**. Further inquiries can be directed to the corresponding author.
